# *rac*-(4a*R*,8a*R*)-2,3-Diphenyl-4a,5,6,7,8,8a-hexa­hydro­quinoxaline

**DOI:** 10.1107/S1600536808016103

**Published:** 2008-06-07

**Authors:** Fang Chen, Heng-Yun Ye

**Affiliations:** a Ordered Matter Science Research Center, College of Chemistry and Chemical Engineering, Southeast University, Nanjing 210096, People’s Republic of China

## Abstract

The structure of the title racemic compound, C_20_H_20_N_2_, shows close similarity to that of the enanti­omerically pure (4a*R*,8a*R*)-2,3-diphenyl-4a,5,6,7,8,8a-hexa­hydro­quinoxaline [Wang & Ye (2008 ▶). *Acta Cryst.* E**64**, o359–o359]. The similarity applies to the unit-cell parameters as well as to the packing of the constituent mol­ecules. Similar packing is conditioned by a lack of directed inter­molecular inter­actions such as hydrogen bonds in either structure.

## Related literature

For examples of the synthesis of non-centrosymmetric solid materials by the reaction of chiral organic ligands and inorganic salts, see: Qu *et al.* (2004 ▶). For geometric parameters of C=N bonds, see: Figuet *et al.* (2001 ▶); Kennedy & Reglinski (2001 ▶). For our previous work regarding the enanti­omerically pure (4a*R*,8a*R*)-2,3-diphenyl-4a,5,6,7,8,8a-hexa­hydro­quin­ox­aline, see: Wang & Ye (2008 ▶).
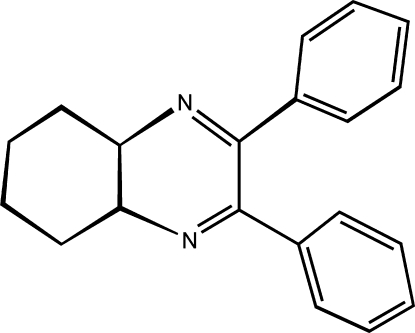


## Experimental

### 

#### Crystal data

C_20_H_20_N_2_
*M* *_r_* = 288.38
Orthorhombic, 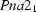

*a* = 15.278 (3) Å
*b* = 18.388 (4) Å
*c* = 5.6638 (11) Å
*V* = 1591.2 (5) Å^3^
*Z* = 4
Mo *K*α radiation
μ = 0.07 mm^−1^
*T* = 293 (2) K
0.25 × 0.15 × 0.10 mm

#### Data collection

Rigaku SCXmini diffractometer
Absorption correction: multi-scan (*CrystalClear*; Rigaku, 2005 ▶) *T*_min_ = 0.831, *T*_max_ = 1.000 (expected range = 0.825–0.993)
16117 measured reflections
2004 independent reflections
1558 reflections with *I* > 2σ(*I*)
*R*_int_ = 0.071

#### Refinement

*R*[*F* ^2^ > 2σ(*F* ^2^)] = 0.047
*wR*(*F* ^2^) = 0.100
*S* = 1.10
2004 reflections
199 parameters
1 restraint
H-atom parameters constrained
Δρ_max_ = 0.13 e Å^−3^
Δρ_min_ = −0.17 e Å^−3^

### 

Data collection: *CrystalClear* (Rigaku, 2005 ▶); cell refinement: *CrystalClear*; data reduction: *CrystalClear*; program(s) used to solve structure: *SHELXS97* (Sheldrick, 2008 ▶); program(s) used to refine structure: *SHELXL97* (Sheldrick, 2008 ▶); molecular graphics: *SHELXTL* (Sheldrick, 2008 ▶); software used to prepare material for publication: *SHELXTL*.

## Supplementary Material

Crystal structure: contains datablocks I, global. DOI: 10.1107/S1600536808016103/fb2093sup1.cif

Structure factors: contains datablocks I. DOI: 10.1107/S1600536808016103/fb2093Isup2.hkl

Additional supplementary materials:  crystallographic information; 3D view; checkCIF report

## Figures and Tables

**Fig. 1.**
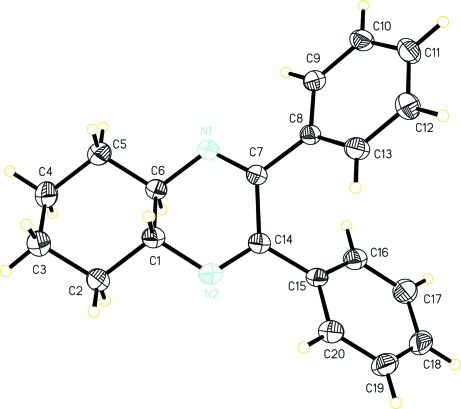
The drawing of one enantiomer with RR configuration of the title compound. The atomic numbering scheme is given. The displacement ellipsoids are drawn at the 30% probability level.

**Fig. 2.**
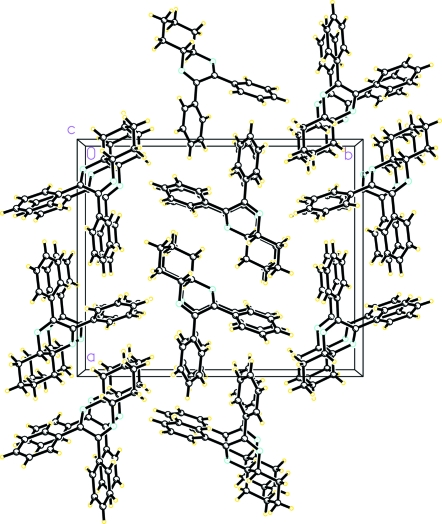
The view of the title compound along the axis c (cf. Fig. 3).

**Fig. 3.**
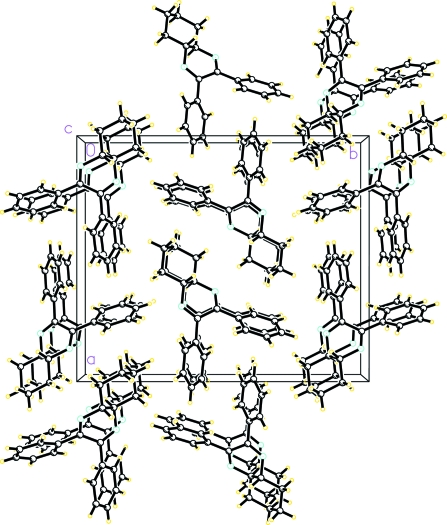
The view of the enantiomerically pure compound (Wang & Ye, 2008) along the axis c (cf. Fig. 2) after suitable transformations (see the comment section).

## supplementary crystallographic information

### Comment

Presence of chiral centres in organic ligands is very important for design and synthesis of noncentrosymmetric or chiral coordination polymers which exhibit desirable physical properties such as a ferroelectric behaviour (Qu *et al.*, 2004). We have recently reported the crystal structure of the enantiomerically pure ligand (4a*R*,8a*R*)-2,3-diphenyl-4a,5,6,7,8,8a-hexahydroquinoxaline (Wang & Ye, 2008). As a part of our ongoing investigations in this field we have determined the crystal structure of the title compound, *rac*-(4a*R*,8a*R*)-2,3- diphenyl-4a,5,6,7,8,8a-hexahydroquinoxaline.

The title compound can be regarded as a derivative of hexahydroquinoxaline by substitution of two H atoms in each of the positions 2 and 3 by the phenyl rings. The heterocyclic ring of the quinoxaline system has a twist-boat configuration, while the cyclohexane ring has a chair configuration. The torsion angle N2—C1—C6—N1 is -58.3 (3)°. The C═N double bonds (C7═N1, 1.272 (2) Å; C14═N2, 1.279 (2) Å) are in the range of 1.27–1.38 Å that have been found in other Schiff base complexes (Figuet *et al.*, 2001; Kennedy & Reglinski, 2001; Wang & Ye, 2008). C7, C14 show typical *sp*^2^ geometry environment. Comparing the bond angles around *sp*^2^ N atoms (N1, N2) with those around the *sp*^2^ C atoms (C7, C14), the latter are somewhat more close to 120°. N1C8C7C14 and N2C15C14C7 are almost coplanar with the mean deviations equal to 0.0119 and 0.0052 Å, respectively. The angle (29.76 (14)°) between the planes of N1C7C8C14 and N2C7C14C15 is very close to that (29.65 (14)°) in the enantiomerically pure compound (4a*R*,8a*R*)-2,3-diphenyl-4a,5,6,7,8,8a-hexahydroquinoxaline (Wang & Ye, 2008). The angle (64.3 (1)°) between both phenyl rings in the title structure equals within the precision of the experiments to that (64.3 (1)°) of the enantiomerically pure compound (4a*R*,8a*R*)-2,3-diphenyl-4a,5,6,7,8,8a-hexahydroquinoxaline.

The title racemic compound crystallizes in the space group of *Pna*2_1_. Figs. 2 and 3 contain the respective views of the unit cells of the title compound and its enantiomerically pure counterpart (Wang & Ye, 2008)). [The enantiomerically pure structure in Fig. 3 has been obtained by the following transformations of the published data (Wang & Ye, 2008): (a,b,c)_2_ = (a,b,c)_1_(0 1 0/0 0 1/1 0 0) followed by the shift of the origin by -1/4 1/2 -1/4 with the corresponding change in the translational parts of the symmetry operators. (0 1 0/0 0 1/1 0 0) is the transformation matrix where each triplet of the numbers corresponds to its row.]

In spite of the fact that a half of the molecules in the title structure are the opposite enantiomers (Fig. 2) in contrast to the structure composed of the enantiomers of one kind in Wang & Ye (2008) (Fig. 3) both structures look alike when wieved along the shortest unit-cell axis. It can not be excluded that both enantiomers form solid solutions in some composition interval. The experiments that would confirm the hypothesis about the formation of the solid solutions are going to be carried out in near future. The melting point of the enantiomerically pure structure (Wang & Ye, 2008) is 194–198°C.

(Note: The setting *P*2_1_*nb* is directly related to that of the reported structure of the enantiomerically pure compound (Wang & Ye, 2008). In the setting *P*2_1_*nb* the unit cell axes are ordered according to their length from the minimal to the maximal.)

### Experimental

*rac*-(1*R*,2*R*/1*S*,2*S*)-diaminocyclohexane was obtained from Adrich. The title compound was prepared by an analogous procedure to that regarding the enantiomerically pure (4a*R*,8a*R*)-2,3-diphenyl-4a,5,6,7,8,8a-hexahydroquinoxaline (Wang & Ye, 2008) using *rac*-1,2-diaminocyclohexane instead of (-)-(1*R*,2*R*)-diaminocyclohexane. Yellow block-like crystals, suitable for X-ray analysis, were obtained by slow evaporation of the ethanol solution of the crude product.

### Refinement

All the H atoms were discernible in difference electron-density map. Nevertheless, they were placed to the idealed positons and refined in a riding atom approximation constrainsts as following: C_methine_—H_methine_ = 0.98; C_methylene_—H_methylene_ = 0.97; C_aryl_—H_aryl_ = 0.93 Å; *U*_iso_H = 1.2 *U*_eq_C in all the cases. In the absence of significant resonant scattering effects, 1639 Friedel pairs were merged.

### Crystal data

**Table:**

C_20_H_20_N_2_	*D*_x_ = 1.204 Mg m^−^^3^
*M**_r_* = 288.38	Melting point = 447–453 K
Orthorhombic, *P**n**a*2_1_	Mo *K*α radiation, λ = 0.71073 Å
Hall symbol: P 2c -2n	Cell parameters from 13431 reflections
*a* = 15.278 (3) Å	θ = 3.3–27.5°
*b* = 18.388 (4) Å	µ = 0.07 mm^−^^1^
*c* = 5.6638 (11) Å	*T* = 293 K
*V* = 1591.2 (5) Å^3^	Block, pale yellow
*Z* = 4	0.25 × 0.15 × 0.10 mm
*F*(000) = 616	

### Data collection

**Table:**

Rigaku SCXmini diffractometer	2004 independent reflections
Radiation source: fine-focus sealed tube	1558 reflections with *I* > 2σ(*I*)
graphite	*R*_int_ = 0.071
Detector resolution: 13.6612 pixels mm^-1^	θ_max_ = 27.5°, θ_min_ = 3.5°
ω scans	*h* = −19→19
Absorption correction: multi-scan (*CrystalClear*; Rigaku, 2005)	*k* = −23→23
*T*_min_ = 0.831, *T*_max_ = 1.000	*l* = −7→7
16117 measured reflections	

### Refinement

**Table:**

Refinement on *F*^2^	Primary atom site location: structure-invariant direct methods
Least-squares matrix: full	Secondary atom site location: difference Fourier map
*R*[*F*^2^ > 2σ(*F*^2^)] = 0.047	Hydrogen site location: difference Fourier map
*wR*(*F*^2^) = 0.100	H-atom parameters constrained
*S* = 1.10	*w* = 1/[σ^2^(*F*_o_^2^) + (0.0344*P*)^2^ + 0.1949*P*] where *P* = (*F*_o_^2^ + 2*F*_c_^2^)/3
2004 reflections	(Δ/σ)_max_ < 0.001
199 parameters	Δρ_max_ = 0.13 e Å^−^^3^
1 restraint	Δρ_min_ = −0.17 e Å^−^^3^
80 constraints	

### Special details

**Table:**

Geometry. All e.s.d.'s (except the e.s.d. in the dihedral angle between two l.s. planes) are estimated using the full covariance matrix. The cell e.s.d.'s are taken into account individually in the estimation of e.s.d.'s in distances, angles and torsion angles; correlations between e.s.d.'s in cell parameters are only used when they are defined by crystal symmetry. An approximate (isotropic) treatment of cell e.s.d.'s is used for estimating e.s.d.'s involving l.s. planes.
Refinement. Refinement of *F*^2^ against ALL reflections. The weighted *R*-factor *wR* and goodness of fit *S* are based on *F*^2^, conventional *R*-factors *R* are based on *F*, with *F* set to zero for negative *F*^2^. The threshold expression of *F*^2^ > 2µ(*F*^2^) is used only for calculating *R*-factors(gt) *etc*. and is not relevant to the choice of reflections for refinement. *R*-factors based on *F*^2^ are statistically about twice as large as those based on *F*, and *R*- factors based on ALL data will be even larger.

### Fractional atomic coordinates and isotropic or equivalent isotropic displacement parameters (Å^2^)

**Table:**

	*x*	*y*	*z*	*U*_iso_*/*U*_eq_	
C1	0.59529 (17)	0.36737 (13)	0.4260 (5)	0.0432 (6)	
H1A	0.5643	0.3988	0.5377	0.052*	
C2	0.55215 (16)	0.29231 (13)	0.4296 (6)	0.0531 (7)	
H2A	0.5849	0.2594	0.3290	0.064*	
H2B	0.5533	0.2731	0.5890	0.064*	
C3	0.45847 (19)	0.29648 (16)	0.3444 (7)	0.0626 (9)	
H3A	0.4241	0.3243	0.4566	0.075*	
H3B	0.4341	0.2478	0.3366	0.075*	
C4	0.4523 (2)	0.33176 (15)	0.1036 (7)	0.0666 (10)	
H4A	0.3913	0.3367	0.0596	0.080*	
H4B	0.4805	0.3008	−0.0123	0.080*	
C5	0.49552 (18)	0.40639 (14)	0.1012 (7)	0.0572 (8)	
H5A	0.4938	0.4262	−0.0575	0.069*	
H5B	0.4635	0.4391	0.2041	0.069*	
C6	0.58925 (16)	0.40112 (12)	0.1829 (5)	0.0413 (6)	
H6A	0.6214	0.3702	0.0718	0.050*	
C7	0.69908 (15)	0.47944 (12)	0.3141 (5)	0.0362 (6)	
C8	0.73733 (14)	0.55333 (12)	0.3446 (5)	0.0362 (6)	
C9	0.72510 (16)	0.60532 (13)	0.1701 (5)	0.0429 (6)	
H9A	0.6967	0.5929	0.0307	0.051*	
C10	0.75498 (16)	0.67548 (14)	0.2028 (6)	0.0482 (7)	
H10A	0.7464	0.7100	0.0849	0.058*	
C11	0.79687 (17)	0.69479 (14)	0.4061 (6)	0.0514 (7)	
H11A	0.8164	0.7423	0.4269	0.062*	
C12	0.81003 (18)	0.64389 (14)	0.5800 (6)	0.0532 (7)	
H12A	0.8391	0.6568	0.7180	0.064*	
C13	0.78012 (17)	0.57348 (13)	0.5501 (5)	0.0453 (7)	
H13A	0.7888	0.5394	0.6690	0.054*	
C14	0.73617 (15)	0.41439 (13)	0.4428 (5)	0.0389 (6)	
C15	0.83132 (16)	0.40920 (13)	0.4971 (5)	0.0397 (6)	
C16	0.89364 (17)	0.43624 (14)	0.3435 (6)	0.0484 (7)	
H16A	0.8761	0.4596	0.2058	0.058*	
C17	0.98203 (18)	0.42881 (15)	0.3928 (7)	0.0583 (8)	
H17A	1.0234	0.4468	0.2875	0.070*	
C18	1.0086 (2)	0.39503 (15)	0.5962 (7)	0.0592 (9)	
H18A	1.0680	0.3903	0.6296	0.071*	
C19	0.94719 (19)	0.36810 (15)	0.7508 (6)	0.0577 (8)	
H19A	0.9652	0.3455	0.8894	0.069*	
C20	0.85886 (18)	0.37447 (14)	0.7016 (6)	0.0510 (7)	
H20A	0.8178	0.3554	0.8058	0.061*	
N1	0.63098 (13)	0.47350 (10)	0.1870 (4)	0.0419 (5)	
N2	0.68676 (14)	0.36140 (11)	0.5019 (5)	0.0475 (6)	

### Atomic displacement parameters (Å^2^)

**Table:**

	*U*^11^	*U*^22^	*U*^33^	*U*^12^	*U*^13^	*U*^23^
C1	0.0398 (14)	0.0371 (14)	0.0528 (17)	0.0033 (11)	0.0011 (13)	0.0030 (13)
C2	0.0423 (15)	0.0418 (15)	0.075 (2)	0.0004 (11)	0.0043 (16)	0.0103 (16)
C3	0.0468 (17)	0.0485 (17)	0.092 (3)	−0.0020 (13)	−0.0008 (17)	0.0044 (18)
C4	0.0511 (17)	0.0546 (18)	0.094 (3)	−0.0083 (14)	−0.0200 (19)	−0.0023 (19)
C5	0.0517 (18)	0.0482 (17)	0.072 (2)	0.0002 (13)	−0.0168 (16)	0.0042 (16)
C6	0.0405 (14)	0.0346 (13)	0.0488 (16)	0.0011 (10)	−0.0028 (13)	−0.0006 (13)
C7	0.0373 (13)	0.0325 (13)	0.0386 (13)	0.0001 (10)	−0.0002 (12)	0.0016 (11)
C8	0.0330 (13)	0.0325 (12)	0.0430 (14)	0.0020 (10)	0.0019 (12)	0.0010 (12)
C9	0.0396 (14)	0.0402 (14)	0.0488 (17)	0.0032 (11)	−0.0021 (13)	0.0051 (13)
C10	0.0448 (14)	0.0376 (14)	0.0622 (18)	0.0005 (11)	0.0078 (16)	0.0099 (14)
C11	0.0486 (16)	0.0362 (14)	0.069 (2)	−0.0068 (12)	0.0058 (16)	−0.0051 (15)
C12	0.0586 (18)	0.0447 (16)	0.0564 (19)	−0.0031 (13)	−0.0055 (15)	−0.0093 (15)
C13	0.0526 (16)	0.0373 (14)	0.0461 (17)	0.0031 (12)	−0.0030 (14)	0.0015 (12)
C14	0.0399 (14)	0.0354 (13)	0.0413 (14)	0.0026 (11)	−0.0009 (12)	0.0014 (12)
C15	0.0413 (14)	0.0286 (12)	0.0492 (16)	0.0052 (11)	−0.0062 (13)	0.0004 (11)
C16	0.0467 (15)	0.0428 (15)	0.0557 (18)	0.0070 (12)	0.0001 (14)	0.0034 (14)
C17	0.0424 (16)	0.0504 (17)	0.082 (3)	0.0058 (13)	0.0053 (16)	−0.0001 (18)
C18	0.0454 (17)	0.0453 (16)	0.087 (2)	0.0093 (13)	−0.0155 (17)	−0.0097 (17)
C19	0.0598 (18)	0.0473 (16)	0.066 (2)	0.0118 (15)	−0.0198 (17)	−0.0003 (15)
C20	0.0545 (17)	0.0424 (14)	0.0561 (18)	0.0018 (13)	−0.0059 (16)	0.0058 (14)
N1	0.0428 (12)	0.0337 (10)	0.0493 (13)	0.0000 (9)	−0.0053 (11)	0.0040 (10)
N2	0.0430 (12)	0.0421 (12)	0.0572 (15)	0.0013 (10)	−0.0049 (12)	0.0115 (11)

### Geometric parameters (Å, °)

**Table:**

C1—N2	1.466 (3)	C9—C10	1.381 (3)
C1—C6	1.513 (4)	C9—H9A	0.9300
C1—C2	1.530 (3)	C10—C11	1.364 (4)
C1—H1A	0.9800	C10—H10A	0.9300
C2—C3	1.512 (4)	C11—C12	1.373 (4)
C2—H2A	0.9700	C11—H11A	0.9300
C2—H2B	0.9700	C12—C13	1.384 (3)
C3—C4	1.513 (5)	C12—H12A	0.9300
C3—H3A	0.9700	C13—H13A	0.9300
C3—H3B	0.9700	C14—N2	1.277 (3)
C4—C5	1.522 (4)	C14—C15	1.489 (3)
C4—H4A	0.9700	C15—C16	1.382 (4)
C4—H4B	0.9700	C15—C20	1.388 (4)
C5—C6	1.508 (4)	C16—C17	1.386 (4)
C5—H5A	0.9700	C16—H16A	0.9300
C5—H5B	0.9700	C17—C18	1.370 (5)
C6—N1	1.476 (3)	C17—H17A	0.9300
C6—H6A	0.9800	C18—C19	1.376 (5)
C7—N1	1.270 (3)	C18—H18A	0.9300
C7—C8	1.489 (3)	C19—C20	1.383 (4)
C7—C14	1.511 (3)	C19—H19A	0.9300
C8—C13	1.385 (4)	C20—H20A	0.9300
C8—C9	1.387 (3)		
			
N2—C1—C6	110.8 (2)	C13—C8—C7	121.8 (2)
N2—C1—C2	109.8 (2)	C9—C8—C7	119.6 (2)
C6—C1—C2	110.8 (2)	C10—C9—C8	120.2 (3)
N2—C1—H1A	108.4	C10—C9—H9A	119.9
C6—C1—H1A	108.4	C8—C9—H9A	119.9
C2—C1—H1A	108.4	C11—C10—C9	120.8 (3)
C3—C2—C1	111.0 (2)	C11—C10—H10A	119.6
C3—C2—H2A	109.4	C9—C10—H10A	119.6
C1—C2—H2A	109.4	C10—C11—C12	119.8 (2)
C3—C2—H2B	109.4	C10—C11—H11A	120.1
C1—C2—H2B	109.4	C12—C11—H11A	120.1
H2A—C2—H2B	108.0	C11—C12—C13	120.1 (3)
C2—C3—C4	111.6 (3)	C11—C12—H12A	119.9
C2—C3—H3A	109.3	C13—C12—H12A	119.9
C4—C3—H3A	109.3	C12—C13—C8	120.6 (3)
C2—C3—H3B	109.3	C12—C13—H13A	119.7
C4—C3—H3B	109.3	C8—C13—H13A	119.7
H3A—C3—H3B	108.0	N2—C14—C15	118.3 (2)
C3—C4—C5	111.6 (3)	N2—C14—C7	120.6 (2)
C3—C4—H4A	109.3	C15—C14—C7	121.1 (2)
C5—C4—H4A	109.3	C16—C15—C20	118.8 (2)
C3—C4—H4B	109.3	C16—C15—C14	121.3 (2)
C5—C4—H4B	109.3	C20—C15—C14	119.9 (2)
H4A—C4—H4B	108.0	C15—C16—C17	120.6 (3)
C6—C5—C4	110.5 (2)	C15—C16—H16A	119.7
C6—C5—H5A	109.5	C17—C16—H16A	119.7
C4—C5—H5A	109.5	C18—C17—C16	120.2 (3)
C6—C5—H5B	109.5	C18—C17—H17A	119.9
C4—C5—H5B	109.5	C16—C17—H17A	119.9
H5A—C5—H5B	108.1	C17—C18—C19	119.7 (3)
N1—C6—C5	110.92 (19)	C17—C18—H18A	120.1
N1—C6—C1	109.2 (2)	C19—C18—H18A	120.1
C5—C6—C1	111.3 (2)	C18—C19—C20	120.5 (3)
N1—C6—H6A	108.4	C18—C19—H19A	119.8
C5—C6—H6A	108.4	C20—C19—H19A	119.8
C1—C6—H6A	108.4	C19—C20—C15	120.2 (3)
N1—C7—C8	117.8 (2)	C19—C20—H20A	119.9
N1—C7—C14	120.8 (2)	C15—C20—H20A	119.9
C8—C7—C14	121.3 (2)	C7—N1—C6	116.1 (2)
C13—C8—C9	118.5 (2)	C14—N2—C1	115.4 (2)
			
N2—C1—C6—N1	−58.1 (3)		
